# Automatism: Are we throwing the baby out with the bathwater?

**DOI:** 10.1177/00258024221108554

**Published:** 2022-06-20

**Authors:** Michael D Kopelman

**Affiliations:** King's College London, Institute of Psychiatry, Psychology & Neuroscience, London, UK

**Keywords:** Automatism, concept/definition, clinical medicine/psychiatry, law/legal, dissociation, law commission

## The concept of automatism

Definitions of automatism abound – legal and clinical. In Bratty versus Attorney General for Northern Ireland, 1963 (1AC, 386), Lord Denning defined automatism as ‘an act which is done by the muscles without any control by the mind such as a spasm, a reflex action or a convulsion; or an act done by a person who is not conscious of what he is doing such as an act done while suffering from concussion or while sleepwalking.’ More recently, in R. versus Coley (2013) (EWCA 223), the England and Wales Appeal Court gave this definition: ‘The essence of it is that the movements or actions of the defendant at the material time were wholly involuntary. The better expression is complete destruction of voluntary control … Examples which have been given in the past include the driver attacked by a swarm of bees or a man under hypnosis. ‘Involuntary’ is not the same as ‘irrational’; indeed it needs to be sharply distinguished from it.’ Rumbold and Wasik^1^ made the point that: ‘The term automatism is used in medicine to denote repetitive, stereotyped actions, usually in the context of complex epileptic seizures. Legal automatism encompasses a whole range of more complex actions,….where the accused has no capacity on grounds of…involuntariness.’

Clinically, the presence of amnesia is a necessary, but not sufficient, condition for automatism. Fenwick^2^ defined automatism as ‘an involuntary piece of behaviour over which an individual has no control. The behaviour itself is usually inappropriate to the circumstances, and may be out of character for the individual. It can be complex, coordinated and apparently purposeful and directed, though lacking in judgement. Afterwards, the individual may have no recollection, or only partial and confused memory for his actions.’ Bourget et al.^3^ and Yeo^4^ both argued that involuntariness and lack of control are much more important features of automatism than the precise level of consciousness (or lack of it). Kopelman^5^ adopted an essentially pragmatic, descriptive definition, which was ‘an abrupt change in behaviour in the absence of conscious awareness or memory formation associated with certain, specific clinical disorders.’ These included epilepsy, parasomnias, hypoglycaemia and head injuries (see also^6^).

Various more specific criteria have been proposed for particular clinical conditions. For example, Delgado-Escueta et al.^7^ proposed that an association between epilepsy and violent behaviour required (i) the diagnosis of epilepsy by at least one neurologist with special competence in epilepsy; (ii) epileptic automatisms documented by the history and closed-circuit television and electroencephologram (EEG) monitoring; (iii) the presence of aggression and simultaneous ictal EEG changes demonstrated by video EEG; (iv) aggressive acts characteristic of the individual's habitual seizures; and (v) the offence should be part of the seizure in the judgement of the attesting neurologist Similar criteria have been proposed for establishing an association between post-ictal confusion and violent offending.^8^ In practice, documentation of automatisms by closed-circuit television and EEG monitoring, or aggression with simultaneous changes demonstrated on EEG/video EEG, are usually unavailable in prison inmates. As a result, the diagnosis usually has to be made on the basis of the history, available EEG evidence, and on ‘the balance of probabilities.’

Similarly, in parasomnias, Ebrahim and Fenwick^9^ have specified criteria for the diagnosis of confusional arousals or sleepwalking, when associated with violence. For example, in a confusional arousal: (i) The subject must have been asleep for a sufficient length of time to reach deep sleep (stage 3 or 4); (ii) The stimulus must be sufficient to induce an awakening; (iii) The abnormal behaviour must start immediately on arousal; and (iv) Usually the episode is short, a matter of a few minutes, but may be lengthened if alcohol is implicated. Where parasomnia is claimed to account for abnormal behaviour on (for example) an aircraft precipitated by alcohol and/or medication (as in some recent cases), there should be evidence of previous parasomnia and/or abnormal arousals on polysomnography. The problem, as the law stands, is that an externally precipitated (‘sane’) automatism does not require expert opinion.

While Scottish law is similar to English law in this matter, there appears to be considerable variability across international jurisdictions. In 2008, the U.S. State of Indiana Court of Appeal ruled (Roger J.Schlatter v. State of Indiana): ‘Automatism is behavior performed in a state of mental unconsciousness apparently occurring without will, purpose, or reasoned intention … a person … capable of action … not conscious of what he/she is doing. Automatism manifests itself in a range of conduct, including somnambulism, hypnotic states, fugue, metabolic disorders, epilepsy and other convulsions, or reflexes.’ Thus, this definition incorporates both metabolic and psychological disorders. In Canada, automatism from somnambulism or epilepsy results in a person being found ‘not criminally responsible because of mental disorder.’ He/she then has to be reviewed within 90 days with regard to determining an appropriate psychiatric ‘disposal’. In this, epileptic automatisms or parasomnias are treated the same as psychoses, delirium tremens, dissociative states, battered women's syndrome and panic reactions, but differently from self-induced alcoholic intoxication. Similarly, in Sweden and Denmark, a neurological or psychiatric finding affects ‘disposal’ or sentencing.^10^

In England and Wales, the Law Commission^11^ proposed a new defence of ‘not criminally responsible by reason of a recognised medical condition,’ which might be a mental or physical disorder. For this to apply, the defendant must ‘wholly’ lack the relevant criminal capacity because of a lack of control, lack of understanding that the act was wrong, or inability to form a rational judgement. This defence would not be available if the lack of capacity were self-inflicted, as in alcohol or substance intoxication. ‘Sane automatism’ would be reserved for those rare cases where the behaviour was not the result of a medical condition – for example, a reflex action. This would bring the law in England and Wales more into line with that in Canada, except that the burden of proof (for both medical and non-medical automatism) would be on the prosecution, unlike Canada (where it is on the defence).^12^ This defence would be available both in Crown Court and in Magistrates’ Courts, and possible ‘disposals’ would range from a hospital order to an absolute discharge.

## Is there a place for ‘psychological blow automatism’?

What remains unresolved is the place of ‘psychological blow automatism’ or ‘dissociative automatism,’^4,13–15^ a matter on which there are important differences across international jurisdictions. Such verdicts are rare in England and Wales, with very occasional exceptions. For example, in the case of R. versus T, 1990 (Crim LR 256), T had been raped 3 days before participating in an armed robbery: the Crown Court ruled that the defence of non-insane automatism was available as a consequence of a dissociative state and PTSD, although the jury rejected this defence and convicted her. In the case of R. versus Roach, 2001 (EWCA Crim 3217), the Court of Appeal ruled that ‘automatism of psychogenic type’, secondary to personality disorder and the combination of alcohol and medications, should have been put to the jury. Such cases appear to be more common in Australia, where evidence of dissociation has led to ‘some questionable acquittals’.^14^ The Canadian Supreme Court ruled that claims of ‘psychological blow automatism’ required ‘an extremely shocking trigger … to establish that a normal person might have reacted to the trigger by entering an automatistic state …’. Nevertheless, commonplace issues such as ‘a traumatic marriage breakdown’ have been accepted as ‘sane automatism’ in Australia and ‘insane automatism’ in Canada.^14^

Bourget et al.^3^ Yeo^4^ and McSherry^14^ have all argued that a lack of volition/control, rather than the level of consciousness, is the critical factor in determining ‘automatism’, with McSherry^14^ positing a continuum across volitional, dissociative and automatic behaviours. But there is, surely, a qualitative difference between those disorders in which voluntary action is ‘wholly’ disrupted by a disturbance of consciousness (e.g. post-epileptic confusion, parasomnias, hypoglycaemia) and those in which volition is merely compromised in the absence of any disturbance of consciousness (sudden shock, PTSD, traumatic marital breakdown)?

Some of the inconsistencies which have arisen, both within and between different jurisdictions, result from the failure to make this important distinction. The concept of medical ‘automatism’ should be reserved for those cases in which volition is disrupted as a direct result of a disturbance of conscious awareness (in conditions such as epilepsy, parasomnias, hyper- or hypoglycaemia, head injury; compare^5^). Problems arise because, in many jurisdictions, the notion of ‘diminished responsibility/competency’ is limited to homicide cases. If ‘diminished responsibility’ were to apply to other offences, the temptation to broaden the concept of ‘automatism’ to include psychological shock or ‘blow’ would no longer exist.

## Conclusion

This raises the question of whether the proposed Law Commission reforms in England and Wales potentially resolve this issue, or throw out the baby with the bathwater. While good arguments can be made for a separate mental disorder defence, the door is now potentially open for a ‘recognised medical condition’, such as ‘adjustment disorder’ or ‘acute stress reaction’, to be treated in the same way as epilepsy, parasomnia, or hypoglycaemia, which will give rise to endless arguments about whether capacity was ‘wholly lacking’ or merely ‘substantially impaired’. The real problem is with the conglomerate term ‘recognised medical condition’, which avoids any attempt to distinguish between (i) those neurological conditions in which volition is traduced by a disturbance of consciousness and (ii) those psychiatric disorders in which volition is merely compromised in the absence of any disruption of conscious awareness. While giving the impression of deftly bypassing this issue, the proposed reforms may, in fact, compound the difficulties that courts will face.
